# Large Scale Analysis of Phenotype-Pathway Relationships Based on GWAS Results

**DOI:** 10.1371/journal.pone.0100887

**Published:** 2014-07-09

**Authors:** Aharon Brodie, Oholi Tovia-Brodie, Yanay Ofran

**Affiliations:** 1 The Goodman Faculty of Life Sciences, Nanotechnology Building, Bar Ilan University, Ramat Gan, Israel; 2 Department of Cardiology, Tel-Aviv Sourasky Medical Center, Tel-Aviv, Israel; University of Illinois at Chicago, United States of America

## Abstract

The widely used pathway-based approach for interpreting Genome Wide Association Studies (GWAS), assumes that since function is executed through the interactions of multiple genes, different perturbations of the same pathway would result in a similar phenotype. This assumption, however, was not systemically assessed on a large scale. To determine whether SNPs associated with a given complex phenotype affect the same pathways more than expected by chance, we analyzed 368 phenotypes that were studied in >5000 GWAS. We found 216 significant phenotype-pathway associations between 70 of the phenotypes we analyzed and known pathways. We also report 391 strong phenotype-phenotype associations between phenotypes that are affected by the same pathways. While some of these associations confirm previously reported connections, others are new and could shed light on the molecular basis of these diseases. Our findings confirm that phenotype-associated SNPs cluster into pathways much more than expected by chance. However, this is true for <20% (70/368) of the phenotypes. Different types of phenotypes show markedly different tendencies: Virtually all autoimmune phenotypes show strong clustering of SNPs into pathways, while most cancers and metabolic conditions, and all electrophysiological phenotypes, could not be significantly associated with any pathway despite being significantly associated with a large number of SNPs. While this may be due to missing data, it may also suggest that these phenotypes could result only from perturbations of specific genes and not from other perturbations of the same pathway. Further analysis of pathway-associated versus gene-associated phenotypes is, therefore, needed in order to understand disease etiology and in order to promote better drug target selection.

## Introduction

### Molecular pathways can assist in revealing disease underpinnings

Genome-wide association studies (GWAS) are a major tool in unraveling genome-phenome relationships. They typically associate a single-nucleotide polymorphism (SNP) with a phenotype with a certain probability, but the molecular relationship between that genomic variation and the phenotype is not apparent in most cases. Attempting to better understand the molecular basis of a phenotype, studies often identify the pathways to which the genes that may be linked to the SNPs belong. However, to establish the association of the phenotype with a pathway, one needs to show that phenotype associated SNPs tend to fall within that pathway more than expected by chance [1]. Analysis of gene sets - defined based on known pathways, GO ID numbers, or other criteria - has been used to bolster results of expression microarrays [2]–[5]. Recently it has also been applied to analyzing GWAS with specific focus on pathways [6], [7]. Results of GWAS have been increasingly used in an attempt to determine which pathways are associated with a certain disease or phenotype [8]. For example, attempts to use GWAS to unravel the molecular basis of major depressive disorder (MDD) have identified only a relatively small number of associated SNPs. Pathway analysis, however, has shown that these SNPs could be used to statistically establish a connection between MDD and inflammatory and immune response [9].

### Are there phenotypes that could not be associated with pathways based on GWAS?

This approach has been applied to GWAS results to identify phenotype-related pathways in various diseases including glioblastoma [10], breast cancer [11], multiple sclerosis [12], Parkinson’s disease [13], autoimmune diseases [14], and other phenotypes [8]. The underlying assumption in such studies is that a given phenotype may be the result of different perturbations of the same pathway. Thus, SNPs that are associated with that phenotype may affect different genes in the same pathway. However, it is conceivable that some phenotypes could be the result of very specific sets of variations that affect some genes but not others, in which case the phenotype-associated SNPs will not cluster into pathways. To our knowledge, the clustering of phenotype-associated SNPs into known pathways has not been assessed systematically on a large scale to determine whether, and to what extent, such clustering occurs in different complex phenotypes. To assess this question, one needs to establish a framework to explore the relationship between phenotypes, SNPs, genes and pathways. Such a framework will also help map pathways to phenotypes systematically in an unsupervised manner, based solely on GWAS results.

### Disease-disease associations based on common molecular basis

Diseases are commonly classified (e.g. in the International Classification of Diseases (ICD)) according to clinical, pathological or epidemiological criteria. However, several studies suggested that classifying diseases according to their common molecular basis could reveal new disease-disease associations and may lead to novel diagnostics and therapies [15], [16]. Large-scale analysis of phenotype-SNP-gene-pathway relationships may help reveal novel disease-disease connections based on pathways that are associated with several diseases.

### Analysis of Phenotype-SNP-Gene-pathway associations

To explore the relationships between phenotype associated SNPs and known pathways, we analyzed all available GWAS data in the NHGRI GWAS catalog [4]. For each phenotype that was associated by SNPs with multiple genes, we assessed whether these genes tend to fall within the same pathway. We found that, indeed, SNPs that are related to the same phenotype tend to affect the same pathway significantly more than expected by chance. However, we identified diseases, conditions, and phenotypes, in which such clustering is not observed despite being significantly associated with a large number of SNPs. In the cases where we found that SNPs significantly fall within specific pathways, we revealed hundreds of phenotype-pathway associations, many of them novel. Based on these associations we could identify molecular connections between phenotypes that are linked to the same pathways. The strongest associations we identified showed a robust link between several autoimmune diseases, which connected not only to each other and to diseases that are suspected as autoimmune, but also to nasopharyngeal carcinoma (NPC). On the other hand, we found that while several types of cancers showed significant associations to pathways, rarely did two types of cancer associate with the same pathway.

## Results

### Clustering of phenotype associated SNPs into pathways is extremely significant

**Figure 1:**
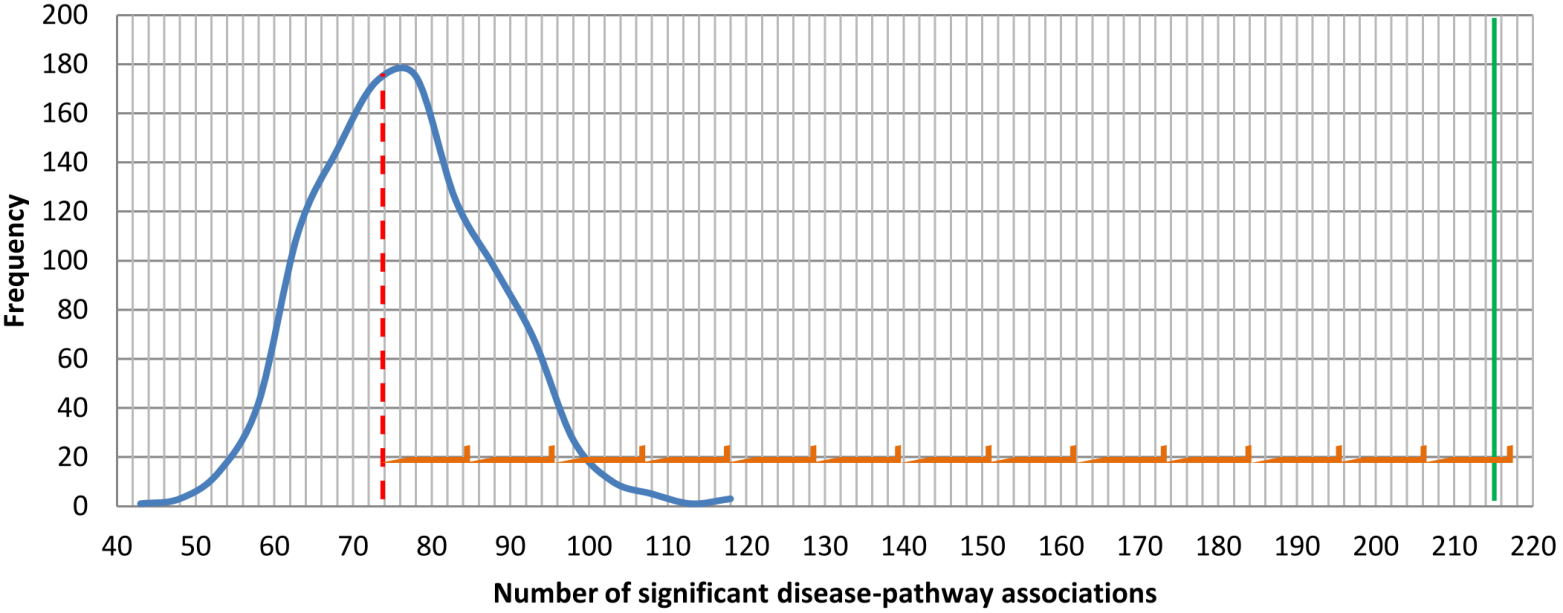
Number of significant phenotype-pathway associations. Curve depicts the distribution of significant phenotype-pathway associations through 1,000 instances of multiple testing runs. X-axis represents the number of phenotype-pathway associations, while the Y-axis represents the frequency. The solid line depicts the real number of phenotype-pathway associations (216), while the dashed line depicts the median of all multiple testing runs (74 phenotype-pathway associations). Any value above the dotted line is significant (0.05). The observed number of phenotype-pathway associations is 12.7 standard deviations above the mean.

We extracted 368 diseases, conditions, and phenotypes for which there are GWAS with significant SNPs. Of those, however, only 213 had ≥2 SNPs that could be associated with a gene (the rest had either none or only one SNP in, or in the vicinity of, a coding region). Thus, only for these 213 phenotypes we were able to assess the clustering of SNPs into pathways. The average number of SNPs per phenotype for these 213 phenotypes was 9.6 (SD = 12.6). For 70 of these testable 213 phenotypes we found 216 significant phenotype-pathway associations. This is extremely significant with respect to what we expect by chance (p-value <10^−5^, resampling test, see Figure 1).

### More SNPs typically mean more significant associations, but there are exceptions

**Figure 2:**
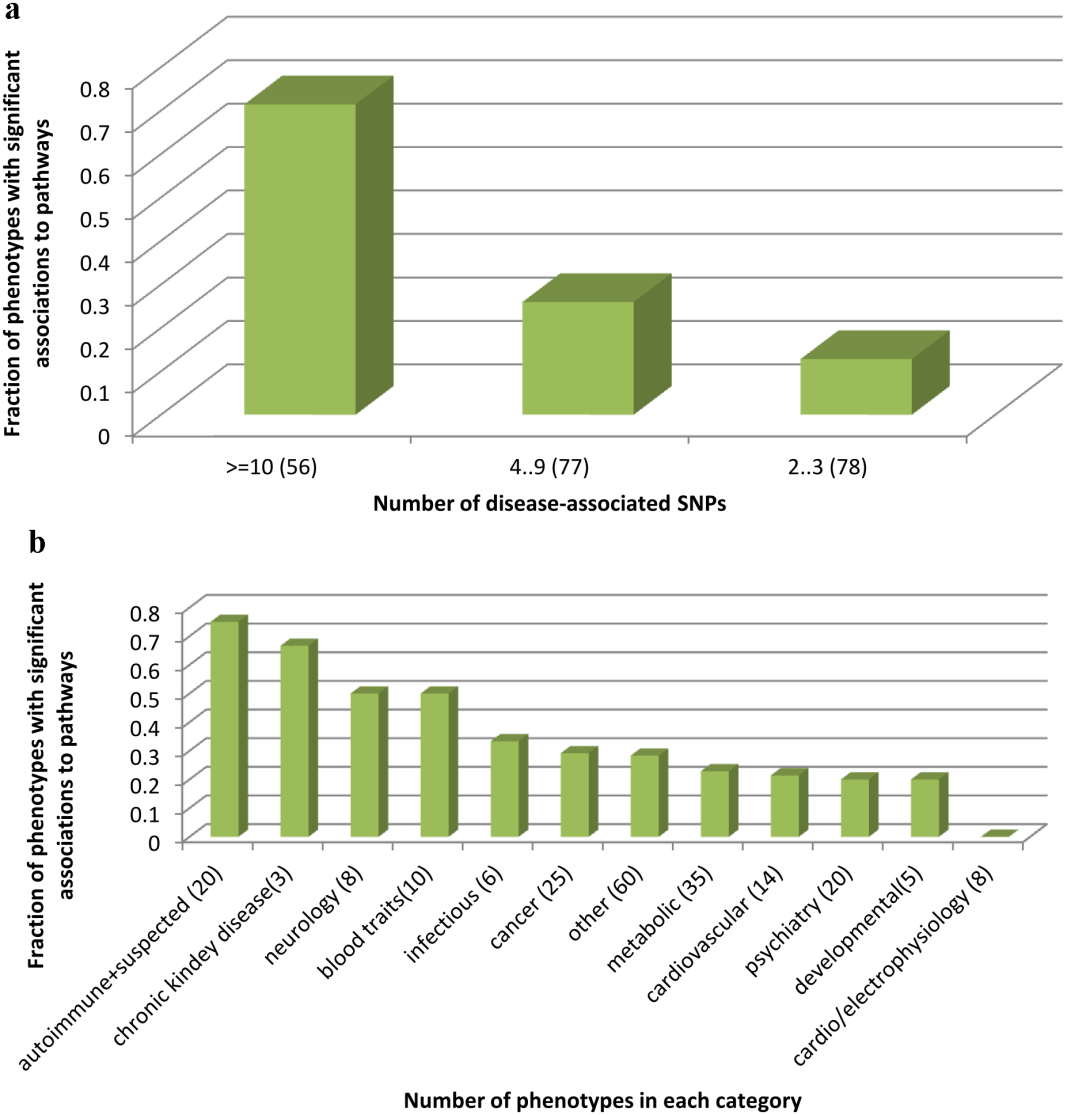
Tendency of different classes of phenotypes to have their SNPs cluster into pathway. X-axis presents phenotypes grouped by categories, while Y-axis represents what fraction of the conditions in this category had at least one significant association with pathways. **A.** Conditions are grouped according to the number of disease SNPs they have (that is SNPs that are significantly associated with the phenotype). Phenotypes for which GWAS found more phenotype SNPs are more likely to be significantly associated with pathways. **B.** Phenotypes are grouped according to types. Autoimmune diseases have a high tendency to cluster to pathways, while other categories, such as psychiatry and metabolic related phenotypes, much less so.

Figure 2 shows how different parameters affect the clustering of SNPs into pathways. In particular, phenotypes for which GWAS found more significant SNPs were more likely to be significantly associated with a pathway (Figure 2A). There are, however, phenotypes with many SNPs that could not be significantly associated with any pathway. For example, cognitive performance or LDL level, each with >30 SNPs identified by GWAS, were not associated with any pathway. On the other hand, esophageal cancer that was found, based on GWAS results, to be associated with only two genes, was significantly associated with 2 different pathways: Fatty acid metabolism and Glycolysis/Gluconeogenesis, as both pathways include the two genes. Thus, significant clustering of phenotype SNPs into pathways can also occur with very few genes.

### Autoimmune diseases tend to cluster much more than cancer and other phenotypes

As shown in Figure 2B, certain types of phenotypes tended more strongly than others to be associated with pathways. Most autoimmune diseases (15 of 20) show significant clustering into pathways. Even when GWAS found only a few disease genes for an autoimmune condition they usually affected the same pathways. The exceptions were comorbidities of two autoimmune diseases (e.g. celiac disease and rheumatoid arthritis), for which no significant associations were found. On the other hand, none of the 8 cardiac-electrophysiological phenotypes showed significant clustering of disease genes into pathways despite the fact that several of them had many associated SNPs (e.g. QT interval duration studies had 13 associated genes). As for cancer, 7 of the 25 cancer-related phenotypes show significant clustering into pathways. This number is similar to that of the general category “other”, which lumps together numerous phenotypes that could not be classified into any of the categories we defined. In both categories, some phenotypes with many SNPs showed no clustering while some phenotypes with a few SNPs showed clustering into pathways. For example, upper aerodigestive tract cancers was associated with only three genes based on its SNPs, but these genes significantly clustered into 6 different pathways (note, many genes appear in several pathways. Thus, 3 genes can fall together into 6 different pathways, which strengthen the hypothesis that they are functionally related). Breast cancer, on the other hand, had 10 associated genes that did not significantly cluster into any pathway (some of these 10 genes did fall together into some pathways, but the resampling procedure showed that this may happen often with 10 random genes as well). Metabolic phenotypes were found to associate with pathways more rarely. GWAS typically found less disease genes for metabolic conditions than they did for autoimmune diseases. However, even if we consider only metabolic conditions with ≥10 disease genes, a mere 54% of them could be associated with pathways (compared to 100% for autoimmune and suspected autoimmune phenotypes with ≥10 genes). Neurological and blood-traits related phenotypes show high clustering into pathways, and cardiovascular, psychiatric and developmental conditions show lower levels of clustering than cancers and “others”. The full list of phenotypes and their results is in Table S1.

### Larger cohorts find more associated SNPs, but not always more pathways

**Figure 3:**
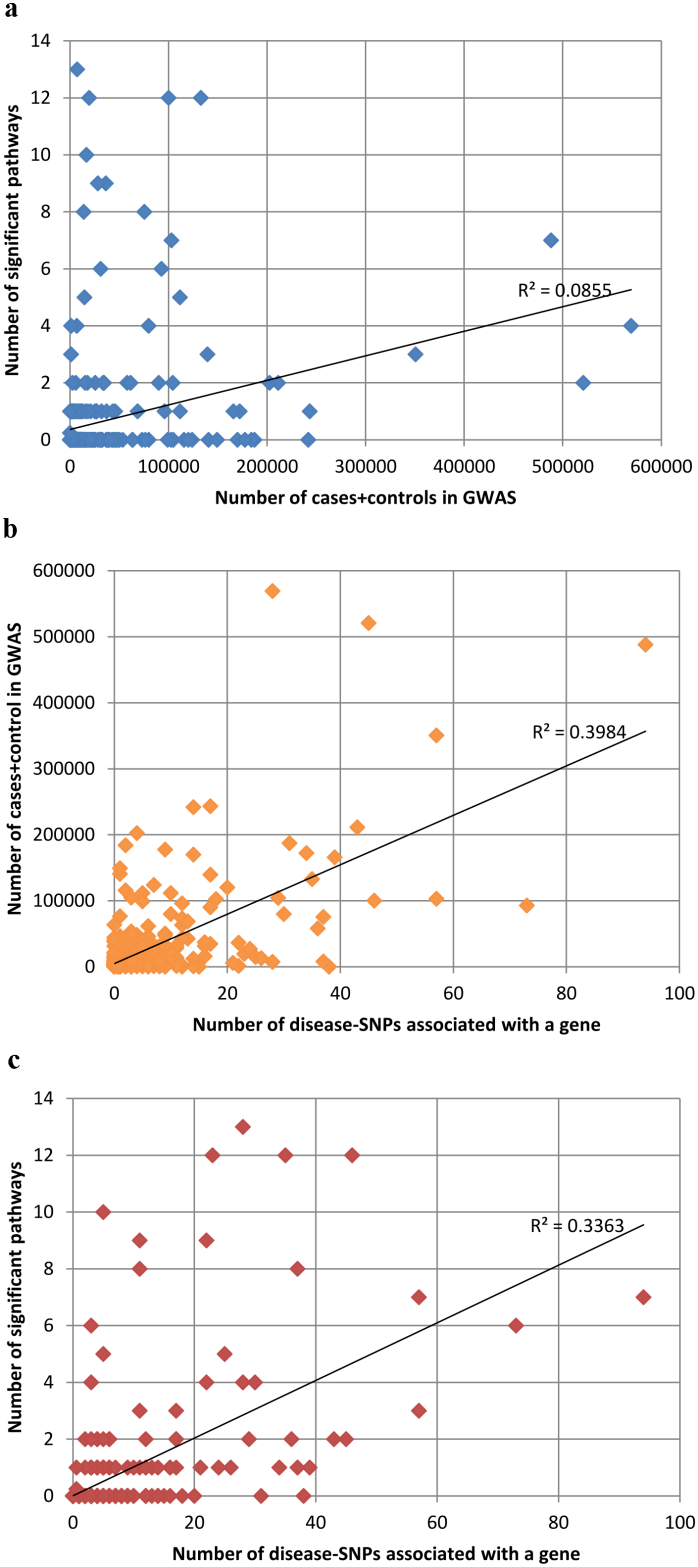
Correlations between number of patients, number of genes and number of pathways. The correlations between the number of individuals in GWAS studies, the number of genes that were found to be significantly associated with the phenotype and the number of pathways significantly associated with the phenotype. **A.** Weak correlation between size of case-control studies and number of pathways significantly associated with phenotypes (Pearson correlation = 0.28). **B.** Correlation between number of phenotype-gene associations and size of case-control studies (Pearson correlation = 0.59). **C.** Correlation between number of phenotype-gene associations and number of significant phenotype-pathway associations (Pearson correlation = 0.58).

Figure 2A shows that phenotypes for which GWAS found more significant SNPs were more likely to be significantly associated with a pathway. One may expect, therefore, that for complex phenotypes, GWAS in which more subjects were tested may identify more associated SNPs, which will reveal more relevant genes, which will then lead to more significant associations of the phenotype to pathways. This expectation is explored in Figure 3. Figure 3A plots the number of patients+controls in all the GWAS of a given phenotype versus the number of pathways with which the phenotype is associated. The correlation is significant but fairly weak (Pearson = 0.28, p-value<0.0001). Notwithstanding, as shown in Figure 3B, the number of subjects is more strongly correlated with the number of associated SNPs (Pearson = 0.59, p-value<0.0001). The number of SNPs is correlated to a similar extent with the number of significant pathways (Figure 3C, Pearson = 0.58, p-value<0.0001). However, since correlation is not transitive, the facts that more patients mean more SNPs, and more SNPs mean more pathways, does not entail that more patients mean more pathways.

### Autoimmune, but not other phenotypes, are associated with the same pathways

**Figure 4:**
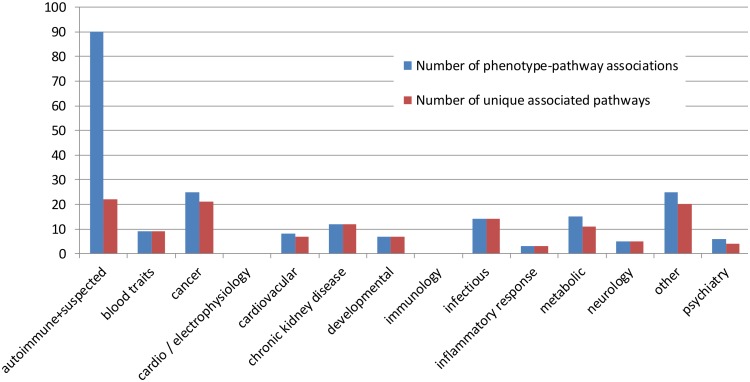
Number of associations and number of unique pathways for different classes of phenotypes. On the Y-axis is the number of pathways and on the X-axis are the classes of phenotypes. In most classes of phenotypes the number of associations found between the phenotypes and pathways is virtually identical to the number of unique pathways associated with the phenotypes in that class. Autoimmune diseases, however, have 90 associations with only 22 pathways.

**Figure 5:**
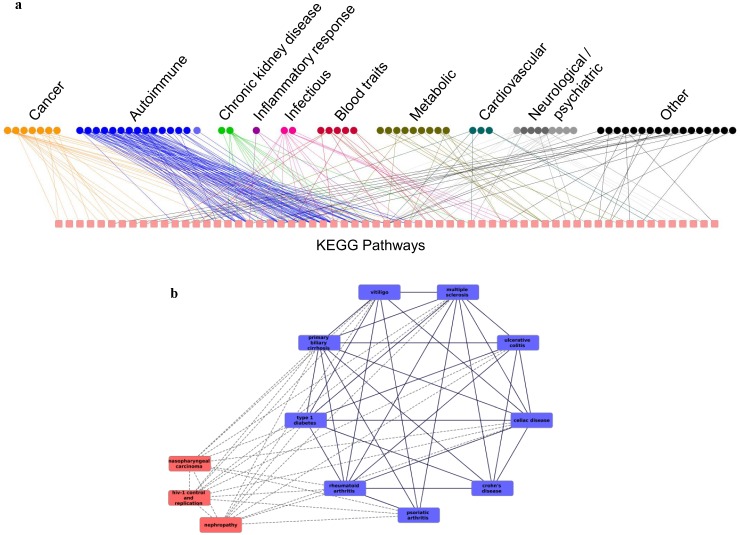
Network representations of phenotype-pathway and phenotype-phenotype associations. **A.** Each node on the top row of this bipartite graph represents a phenotype. Each square on the bottom row represents a pathway. Autoimmune diseases tend to associate with the same pathways while other classes of phenotypes associate with different pathways **B.** Nodes represent phenotypes. An edge indicates that both phenotypes are significantly associated with at least 3 common pathways. Blue nodes are autoimmune phenotypes while red nodes are non-autoimmune. Solid lined links are between two autoimmune phenotypes while dotted lines show links to other phenotypes.

Figure 4 compares the number of phenotype-pathway associations for each group of phenotypes to the number of unique pathways with which they are associated. For most classes of phenotypes, each pathway is associated with only one phenotype from that class. However, for autoimmune diseases, there are 90 different phenotype-pathway associations for only 22 pathways. That is, each pathway is associated, on average, with more than four different autoimmune diseases. This can also be seen in Figure 5A, which shows in a bipartite graph the links between phenotypes and pathways. The autoimmune conditions are linked repeatedly to the same pathways while the other categories of conditions link to different pathways.

### Phenotype-pathway associations highlight molecular connections between phenotypes

**Figure 6:**
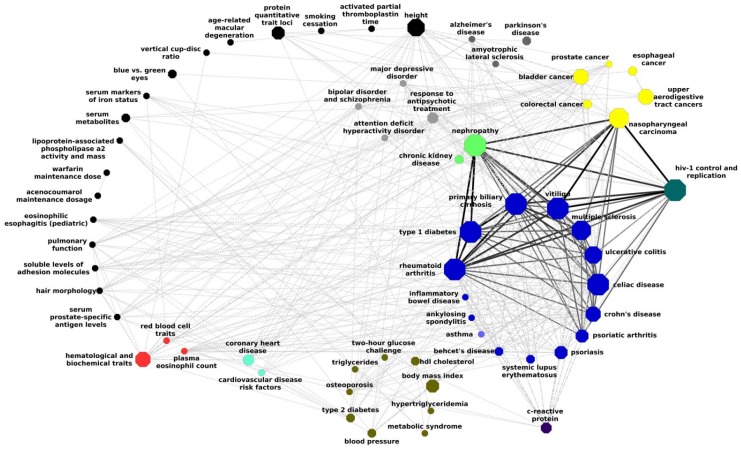
Large scale network representation of phenotype-phenotype associations. This network was created by all 391 phenotype-phenotype associations based on significantly associated phenotype-pathway associations of the entire GWAS dataset.

Based on the significant phenotype-pathway associations, we were able to link phenotypes that are associated with the same pathways to each other. This link indicates that the two phenotypes may be affected by similar molecular mechanisms. The 216 significant phenotype-pathway associations we identified created 391 phenotype-phenotype links between 63 phenotypes. Each of these 391 links (which are, of course, only a small fraction of all possible links) is based on at least two significant phenotype-pathway associations (at least one for each phenotype). The full list of phenotype-phenotype links and their scores is in Table S2. Based on these links it is possible to draw a network of phenotypes where each node is a phenotype and each edge represents a shared molecular basis, manifested in at least one significant disease-phenotype association for each phenotype. Figure 5 presents such a network. In Figure 5B we present a network that is based only on pairs of phenotypes that are associated by at least 3 pathways (that is, by at least 6 significant phenotype-pathway associations, 3 for each phenotype). This network has one large connected component of mainly autoimmune diseases. Interestingly, three phenotypes that are not clearly autoimmune are connected with multiple edges to the rest of the, mostly autoimmune, connected component. They are HIV control and replication, nephropathy and NPC. Importantly, the network is generated automatically in an unsupervised manner without manual interferences or labeling of phenotypes by type. Thus it simply reveals groups of phenotypes that affect individuals with genomic variations in the same pathways. Table 1 presents the KEGG pathways that associate HIV control and replication, nephropathy and NPC to the rest of the network. The connected component is not a clique, that is, not all autoimmune conditions in it share pathways with all other autoimmune diseases, indicating that not all autoimmune diseases could be associated with the same pathways. Moreover, not all autoimmune conditions in the data are included in this connected component. A full network based on all 391 links is presented in Figure 6. It is possible to see that most autoimmune diseases are connected to each other while different types of cancer are very sparsely connected to each other. Out of 7 types of cancers that have significant associations with pathways, only 6 are in this network, indicating that 1 of the cancers was associated with pathways that are not associated with any other phenotype.

**Table 1:** Pathways significantly associated with autoimmune diseases and also with HIV, nephropathy or NPC.

Phenotype	Pathway ID	Number of Phenotype-genes in Pathway	P-value	Pathway name
HIV-1 control and replication	hsa04010	3	0.03195861	MAPK signaling pathway
HIV-1 control and replication	hsa04144	4	0.005693633	Endocytosis
HIV-1 control and replication	hsa04514	3	0.03195861	Cell adhesion molecules (CAMs)
HIV-1 control and replication	hsa04612	4	0.005693633	Antigen processing and presentation
HIV-1 control and replication	hsa04650	5	0.000965442	Natural killer cell mediated cytotoxicity
HIV-1 control and replication	hsa04940	3	0.03195861	Type I diabetes mellitus
HIV-1 control and replication	hsa05320	3	0.03195861	Autoimmune thyroid disease
HIV-1 control and replication	hsa05330	3	0.03195861	Allograft rejection
HIV-1 control and replication	hsa05332	3	0.03195861	Graft-versus-host disease
HIV-1 control and replication	hsa05416	3	0.03195861	Viral myocarditis
Nasopharyngeal carcinoma	hsa04144	3	0.005692787	Endocytosis
Nasopharyngeal carcinoma	hsa04514	5	4.95E-05	Cell adhesion molecules (CAMs)
Nasopharyngeal carcinoma	hsa04612	4	0.00039602	Antigen processing and presentation
Nasopharyngeal carcinoma	hsa04940	4	0.00039602	Type I diabetes mellitus
Nasopharyngeal carcinoma	hsa05320	4	0.00039602	Autoimmune thyroid disease
Nasopharyngeal carcinoma	hsa05330	4	0.00039602	Allograft rejection
Nasopharyngeal carcinoma	hsa05332	4	0.00039602	Graft-versus-host disease
Nasopharyngeal carcinoma	hsa05416	4	0.00039602	Viral myocarditis
Nephropathy	hsa04514	2	0.046744722	Cell adhesion molecules (CAMs)
Nephropathy	hsa04612	2	0.046744722	Antigen processing and presentation
Nephropathy	hsa04672	2	0.046744722	Intestinal immune network for IgA production
Nephropathy	hsa04940	2	0.046744722	Type I diabetes mellitus
Nephropathy	hsa05310	2	0.046744722	Asthma
Nephropathy	hsa05320	2	0.046744722	Autoimmune thyroid disease
Nephropathy	hsa05322	2	0.046744722	Systemic lupus erythematosus
Nephropathy	hsa05330	2	0.046744722	Allograft rejection
Nephropathy	hsa05332	2	0.046744722	Graft-versus-host disease
Nephropathy	hsa05416	2	0.046744722	Viral myocarditis

## Discussion

### Genes affecting a phenotype tend to come from the same molecular pathways

Our results provide statistical evaluation of the general assumption that disease-associated genes cluster into pathways significantly more than randomly selected gene sets of the same size. Figure 1 shows the extent of clustering we see in real GWAS data compared to the clustering we should expect from comparable random sets of genes. Compared to the clustering of random sets of genes into KEGG pathways, the number of significant phenotype-pathway associations we found is 12 standard deviations greater than the mean of 1000 random reshuffles. It is important to note that these assessments are conservative as we ignore the fact that the number of genes that fall into a pathway is typically larger for the real GWAS data than it is for the background random model (see for example the analysis of the association of NPC to KEGG pathway hsa04514 in Figure S1).

These results suggest that for many of the phenotypes, different perturbations of different genes in the same pathway lead to the same phenotype. However, we also find that for most phenotypes, the genes identified by GWAS could not be associated significantly with any pathway. Is this due to a failure in identifying existing phenotype-pathway associations, or is it because these phenotypes are a result of specific genomic variations and could not be caused by alternative perturbations to the same pathway? It is hard to answer this question conclusively. First, a pathway is not a well-defined entity. Second, our knowledge of pathways is partial at best. Many pathways are not yet known and many of the pathways that we used for our analysis may include in reality components of which KEGG curators are not yet aware. Moreover, it is possible that biases in the definitions of pathways in KEGG, to which we are not aware, affected our results (for a discussion on the coverage and accuracy of KEGG see [17]). As knowledge of biological networks grows, it will probably be possible to discover additional phenotype-pathway associations using the same SNP data. Similarly, as more disease SNPs are identified by new GWAS, it will be possible to find more phenotype-associated genes and hence more phenotype-pathway associations even with the currently known pathways. Notwithstanding, in some cases, clustering of the phenotype-associated genes into pathways should not be expected. For example, Mendelian phenotypes may be caused by different variations in one protein, while other perturbations of the same pathway that do not affect that protein will typically not lead to a similar phenotype (note, we considered SNPs to be clustered into one pathway only if they are associated with different genes. Thus, cases in which different perturbations of a single protein lead to the same phenotype were not considered). While our results also show, not surprisingly, that it is more likely to find an underlying pathway for a phenotype if GWAS found more disease genes, we also show that some types of phenotypes cluster into pathways more than others, regardless of the number of disease genes.

Large-scale analysis of the entire Catalog of Published Genome-Wide Association Studies allowed us to explore how different parameters affect disease-gene or disease-pathway associations. We see a moderate positive correlation between the number of patients in a study and the number of genes detected (Fig. 3B). We further show a moderate positive correlation between the number of associated genes and the number of phenotype-pathway associations (Fig. 3C). However, this is not transitive: we found a significant but weak correlation between patient group size and disease-pathways (Fig. 3A), suggesting that a larger patient group only marginally increases the likelihood of finding more associations with pathways. Incidentally, if one focuses on the phenotypes with the largest number of patients (>100.000), none of them could be associated with more than 2 pathways, and most of them could be associated with none. On the other hand, some of the phenotypes that were associated with the highest number of pathways (e.g. 14) were studied on relatively small groups (e.g. <1000).

Various studies have used GWAS to identify phenotype-pathway associations but they have done so only for a small set of diseases or phenotypes [8], [14], [18]–[20]. Many of these studies rely on data from large case-specific studies such as the WTCCC [21], Farmingham Heart Study [22], or the International Cancer Genome Consortium (ICGC) [23]. Our study uses integrated data from all GWAS curated into the NIH GWAS Catalog. This allowed us to explore phenotype etiology on a larger scale, and provide a significantly broader view of phenotype pathway and phenotype-phenotype relationships.

### Many autoimmune diseases are associated with the same set of pathways

The large connected component of mostly autoimmune diseases presented in Figure 5B indicates that these diseases are affected by similar molecular mechanisms. Several autoimmune diseases, such as primary biliary cirrhosis, multiple sclerosis, rheumatoid arthritis, and celiac, associated with pathways which are marked in KEGG as related to immunity, like the pathways “Type 1 Diabetes”, “viral myocarditis”, “graft vs. host disease”, “autoimmune thyroid disease”, “systemic lupus erythematosus”, and “asthma”. Other pathways associated with autoimmune diseases include pathways related to cell adhesion molecules, allograft rejection, antigen processing, cytokine-cytokine receptor interactions, and intestinal immune network, known to be associated with immunological processes. It has been shown that autoimmune responses are involved in transplant rejections [24]. Inflammation, a major factor in many autoimmune disorders, involves cell adhesion molecules that are critical for leukocyte activation, circulation, and localization [25]. The association of autoimmune diseases to viral myocarditis that came up in our analysis is consistent with a previous report [26]. The autoimmune relationships seen here are consistent with the tendency of autoimmune diseases to appear together in patients or in families. This is manifested, for example, in Polyglandular Autoimmune Syndromes, which classify autoimmune disease co-morbidities [27]. Our results demonstrate the genomic basis for these co-morbidities.

### Molecular connection between NPC and autoimmunity

NPC is more common in Southeast Asian regions and to individuals of southern Chinese origin [28]–[30]. It has higher incidence in areas where Chinese-style salted fish is common [31]. This suggests that genetic factors play a major role in NPC, but there are possibly environmental factors that affect its incidence as well [30]. NPC has also been shown to be closely related to EBV infection [32], which may suggest involvement of an abnormal immunological response to this common virus. We found NPC to be significantly associated with 10 immune and autoimmune related pathways, while other cancers in the dataset did not show such strong associations (NPC is also associated with 8 other pathways that are not related to autoimmunity). In particular, NPC is significantly associated with pathways related to cell adhesion molecules, allograft rejection, antigen processing and presentation, autoimmune thyroid disease, graft-versus-host disease, Type I Diabetes, and viral myocarditis. The role of cell adhesion molecules in cancer has been discussed before [33]–[36]. Cell adhesion molecules can influence metastatic potential of tumors by both encouraging and suppressing adhesion. Adhesion is enhanced near primary tumor sites, and is also a key factor in cell migration, e.g. adhesion to venular endothelial cells when migrating across vessel walls. Adhesion is suppressed when cells travel through vessels as well as when they pass through vessel walls into surrounding tissues [37]. This has also been studied specifically in NPC [38], [39]. NPC’s association with antigen presentation and processing (particularly with HLA-related pathways) has been previously discussed [40]. It has been suggested that MHC antigens play a crucial role in tumor development in general [41], but not specifically in NPC. We did not find a link in the literature between NPC and graft-versus-host disease, Type I Diabetes, or viral myocarditis. Possible connections between autoimmune diseases and cancer have been discussed, particularly the association between autoimmunity, inflammation, and cancer development [42]. There is also a known link between cancer immunosuppression and autoimmune diseases [43]. While previous studies suggested that some autoimmune diseases increase the risk for some types of cancer (including e.g. Type 1 Diabetes patients [44]), to our knowledge, NPC was not specifically pointed out to be associated with autoimmune diseases. Our findings are particularly interesting in this context as this association is data-driven, and not hypothesis-driven. It emerges from the GWAS results connecting NPC to autoimmune diseases in multiple highly significant connections. Thus, further investigation of this association is warranted.

### NPC linked to HIV

A link was found between NPC and HIV. It has been shown that incidence of cancer is higher for HIV-infected persons than the general population [45], mainly regarding Kaposi sarcoma and non-Hodgkin lymphoma [46]. However, risk elevation has been shown in various other cancer types, such as leukemia, melanoma, lung and liver [47]. While an increased risk for head and neck cancers (HNC) is mentioned [48], we did not find NPC discussed specifically. Our results indicate a link between autoimmunity, HIV, and NPC. EBV is known to be strongly associated with NPC [49]–[52], as well as HIV [53], [54] and autoimmune diseases [55]. This may suggest a direction for future investigation of these suggested links.

### HIV replication associated with immune and autoimmune pathways

HIV-related SNPs in our dataset are common variants that were found to be phenotypically associated with viral load. The impact of genetic variation on viral replication/viral load has been demonstrated in the past [56]–[58]. We found that viral load is significantly associated with pathways related to antigen processing and presentation, Type 1 Diabetes, and natural killer cell mediated cytotoxicity. Variations related to antigen processing have been shown to affect the course of HIV infection in numerous ways, such as epitope abundance, and cleavage patterns [59]–[61]. Natural killer cells have an important role in HIV control [62]–[64], and HIV’s evasion of these cells has been discussed as well [65]. The cytotoxic effects they have on virus infected cells may be inhibited by overruling the actions of the activating receptors, such as tampering with MHC expression [66]. Although the majority of HIV patients that developed diabetes, developed Type 2 Diabetes [67], it has been reported that autoimmune diabetes (Type 1) develops in some HIV-infected patients after immune restoration during highly active antiretroviral therapy (HAART) [68]. Genetic variations in the associated pathway may be related to this co-morbidity. Immune dysregulation and factors associated with the immunopathology of HIV infection fit the current understanding of autoimmunity [69], [70]. The relationship between HIV and autoimmunity we report may assist in further studying this relationship.

### Different choices may affect some of the results, but the overall trends will remain

Our attempt to assess the clustering of SNPs into known pathways in an unbiased manner forced us to make many choices. For example, KEGG is one of several pathway repositories. It is possible that choosing another repository or combining several such sources would have allowed for the identification of other associations. Our decision to manually group phenotypes by a physician, which went over the published GWAS publications and grouped phenotypes based on pathological and etiological similarities, could have been done in other ways (e.g. using ontological definitions of diseases such as Disease Ontology [71] or Human Phenotype Ontology [72]). This could have led to somewhat different associations. Clearly, each grouping that one may use, disregards some similarities. For example, asthma is categorized as a respiratory system disease in Disease Ontology. We categorized it as a suspected autoimmune disease due to studies suggesting a relationship between asthma and autoimmunity [73], [74] based, in part, on the role of mast cells in the disease [75]–[77]. We put autism under psychiatry, while others may classify it as developmental. Different choices may result in slightly different categories. However, the overall trend will remain. Our results show that grouping phenotypes that have real common denominators reveals which phenotypes have similar behaviour (e.g. autoimmune diseases as compared to the category “other”).

In conclusion, we present a framework for the study of the relationships between phenotypes, SNPs, genes and pathways, and show that phenotype-associated SNPs can reveal novel and unexpected connections between phenotypes and pathways. Applying this framework to additional SNP data using larger sets of pathways may allow for more insights into the molecular basis of diseases and reveal more connections between diseases.

## Methods

### Data

Phenotype-SNP associations were extracted from GWAS data in the NHGRI GWAS catalog (June 2011) [4]. This database contains manually curated entries of published GWAS, in which SNPs were associated with diseases, phenotypes, and traits. We merged different GWAS entries of the same phenotype, which resulted in 368 phenotypes. Gene symbols were taken from Gene names [78], while the genomic locations of SNPs and genes were taken from the UCSC genome browser [79]. Biological pathways and their associated genes were taken from the KEGG pathway database (release 53) [80].

### Grouping phenotypes into categories

Entries in the NHGRI GWAS catalog encompassed 368 phenotypes. Phenotypes were manually grouped into categories by a physician based on pathogenesis. Phenotypes were grouped into 13 categories such as “cancer” and “chronic kidney disease”. Phenotypes not fitting into any group were placed in “others”. The phenotypes and categories can be seen in Table S3. For example, the etiology of cardiovascular diseases is different from cardio/electrophysiology related diseases. Atherosclerotic processes are the basis for cardiovascular diseases, whereas cardio/electrophysiology diseases are mainly based on genetic factors. The difference between neurology and psychiatry is based on the classification of the ICD-10 classification of mental and behavioural disorders [81]. Diseases present in ICD-10 were classified as psychiatric in our research.

### Association of phenotypes to pathways

We defined a SNP as a phenotype SNP if it is associated with the phenotype in the NHGRI GWAS catalog (see Hindorff et al. [4]). Entries are listed in the catalog if they associate with the phenotype with p-values <1e^−5^. We link SNPs to genes as in [82], [83]. The gene in which, or near which [82], [83], a phenotype SNP occurs is defined as a phenotype-associated gene or phenotype gene. To determine whether a pathway is significantly associated with a phenotype we assessed whether the phenotype-associated genes fall within that pathway significantly more than expected at random, based on the number of phenotype-associated genes and the size of the pathways in KEGG [80] (see below).

### Scoring methods

**Figure 7:**
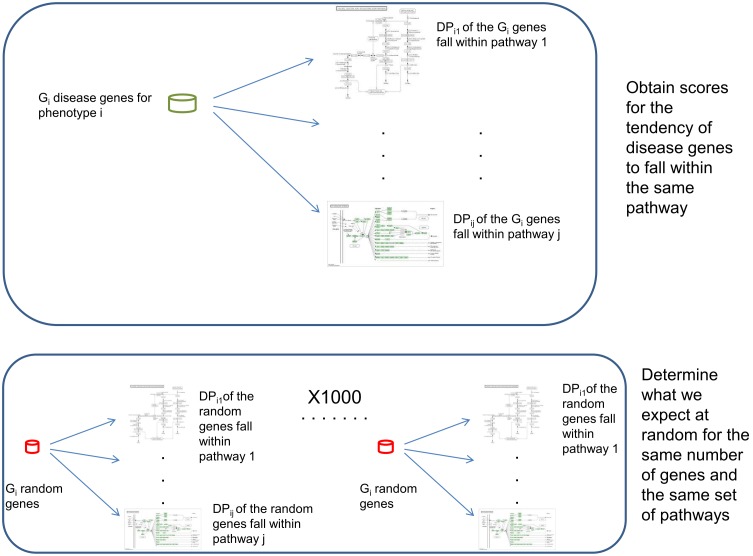
The procedure for assessing significance of phenotype-pathway associations. For phenotype i and we counted how many of the genes associated with it fall within each of 198 KEGG pathways. A pathway was said to be significantly associated with a phenotype if this number was significantly higher than expected by chance. To determine what is expected by chance, we randomly sampled the same number of genes 1,000 times.

To assess significance of a phenotype-pathway association, we defined an association score DP_ij_, for the association between phenotype i and pathway j. DP_ij_ is the number of genes in pathway j that are associated with SNPs of phenotype i. The significance of each association is assessed as follows: G_i_ is the number of disease genes of phenotype i that also appear in any KEGG pathway. We randomly selected G_i_ genes from all genes in all KEGG pathways and calculated DP_ij_ for this random set. That is, for each pathway, we counted how many of the G_i_ random genes fall within that pathway. This was done 1,000 times for each phenotype i, resulting in a set of vectors of scores, which we dub expected random scores (ERS). The vector ERS_ij_ (of 198,000 elements, 1,000 random scores for the clustering of G_i_ genes into each of KEGGs 198 pathways) represents the distribution of DP_ij_ expected at random for the association between phenotype i and pathway j under the null hypothesis that SNPs of phenotype i do not cluster into any pathway. Figure 7 presents this procedure schematically. If the real DP_ij_ ≥95% of scores in ERS_ij_ (i.e. it is within, or higher than, the top 0.05 of the random scores) then the association between phenotype i and pathway j is defined as significant.

In addition, we assessed the number of significant phenotype-pathway associations that are expected at random given the procedure above under the null hypothesis, to account for multiple testing. To this end we repeated the procedure described above 1,000 times for each phenotype versus all pathways, each time with a random set of pseudo phenotype genes, to determine how many significant disease-pathway association we should expect at random given the procedure. This was done as follows:

For each phenotype i, we randomly picked G_i_ genes from KEGG genes as described above. This random set was now defined as the pseudo phenotype genes pG_i_ = G_i_. For these pG_i_ genes we calculated pDP_ij_ as described above for each pathway. Next, for this pseudo phenotype-pathway association we again calculated ERS_ij_ with 1,000 randomly selected G_i_ genes and determined whether the pG_i_ “significantly” cluster into that pathway. We repeated this for each pathway and recorded the number of pathways with which phenotype i is associated. This was performed over all the phenotypes, yielding a number that represents all the significant associations between the pseudo phenotype genes and KEGG pathways.

The above procedure was repeated 1,000 times for each phenotype. The result of these 1,000 simulations gave us the number of phenotype-pathway associations expected at random given the sizes of our data sets.

The approach we have taken here makes no theoretical assumptions regarding the distributions of the statistics and provides us with a genuine assessment of the expected results given our specific datasets under the null hypothesis. Indeed, we took the strictest approach, ignoring distributions of each individual score and factors like the number of genes that were clustered into each pathway. Thus, this approach does not take into account the fact that the real genes tend to cluster more strongly into pathways than the pseudo disease genes. In this sense, it describes the worst-case scenario in terms of the random expectation and exacerbates the burden of proving significance.

### Phenotype-phenotype links

Two phenotypes were linked if they are associated with the same pathway. The strength of the association between two phenotypes could be measured by the number of different pathways with which both of the phenotypes are significantly associated. However, some phenotype-pathway associations are stronger than others. A phenotype-phenotype link is scored using the number of shared pathways between these phenotypes.

Calculations were run in python under Red Hat Linux as well as Microsoft Excel, which was also used to generate the figures.

## Supporting Information

Figure S1 **Association of nasopharyngeal carcinoma to KEGG pathway hsa04514 (cell adhesion molecules).** Curve depicts the amount of randomly selected genes found in each pathway through 1,000 random runs. X-axis represents the number of genes in the pathway, while the Y-axis represents the frequency. The solid line depicts the actual number of NPC genes in pathway hsa04514 (5), while the dashed line depicts the median of all random runs (0). p-value is 0.01E-4. Any value higher than the dotted line is significant (<0.05).
(PDF)

Table S1 **Full list of phenotypes and their results.** Table lists the number of associated SNPs, genes, and pathways for each phenotype.
(XLSX)

Table S2 **Full list of phenotype-phenotype links and their scores.** Table lists phenotype-phenotype links and the number of significant associations to the same pathways.
(XLSX)

Table S3 **Phenotypes grouped into categories.** Table lists phenotypes and categories they were grouped into. Phenotypes not fitting into any of the groups were placed in “other”.
(XLSX)
