# Supplementary material for: Large Scale Analysis of Phenotype-Pathway Relationships Based on GWAS Results
Source: PLoS One. 2014 Jul 9;9(7):e100887. doi: 10.1371/journal.pone.0100887 (PMC4090199; doi:10.1371/journal.pone.0100887)
Supplement: Figure S1 — Association of nasopharyngeal carcinoma to KEGG pathway hsa04514 (cell adhesion molecules). Curve depicts the amount of randomly selected genes found in each pathway through 1,000 random runs. X-axis represents the number of genes in the pathway, while the Y-axis represents the frequency. The solid line depicts the actual number of NPC genes in pathway hsa04514 (5), while the dashed line depicts the median of all random runs (0). p-value is 0.01E-4. Any value higher than the dotted line is significant (<0.05). (PDF) [file pone.0100887.s001.pdf]

Table of contents:

1. Supplementary Figure S1

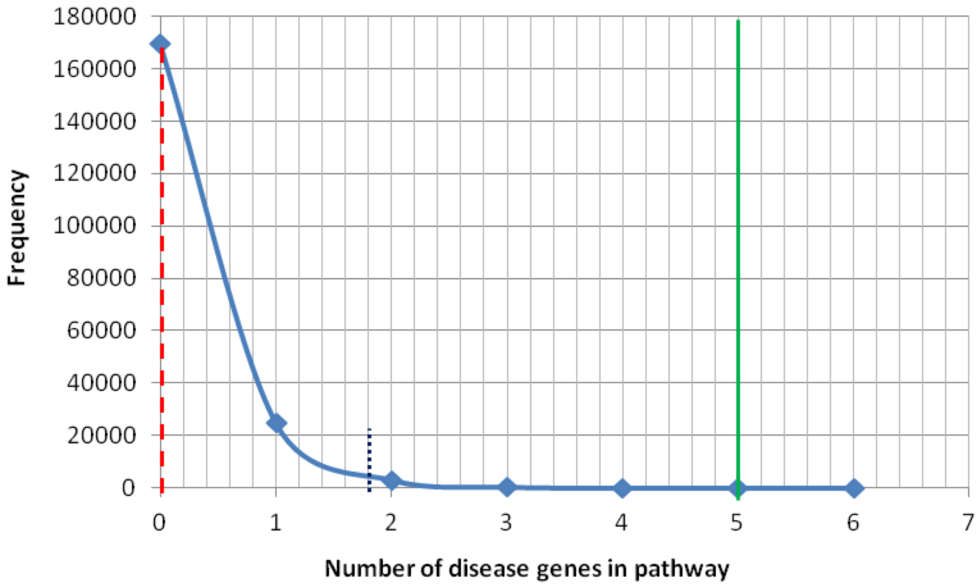

Figure S1: Association of nasopharyngeal carcinoma to KEGG pathway hsa04514 (cell adhesion molecules). Curve depicts the amount of randomly selected genes found in each pathway through 1,000 random runs. X-axis represents the number of genes in the pathway, while the Y-axis represents the frequency. The solid line depicts the actual number of NPC genes in pathway hsa04514 (5), while the dashed line depicts the median of all random runs (0). p-value is 0.01E-4. Any value higher than the dotted line is significant (< 0.05).
